# A small clinical trial of vaporized cannabis for PTSD: suggestive results and directions for future study

**DOI:** 10.1186/s13063-023-07543-2

**Published:** 2023-09-09

**Authors:** Zach Walsh, Ian Mitchell, Kim Crosby, Michelle St. Pierre, Drew DeClerck, Kaye Ong, Philippe Lucas

**Affiliations:** 1 Department of Psychology, The University of British Columbia, Kelowna, BC Canada; 2 Department of Emergency Medicine, The University of British Columbia, Vancouver, BC Canada; 3 Tilray, Nanaimo, BC Canada; 4 BC Cancer, Vancouver, BC Canada; 5 Social Dimensions of Health, The University of Victoria, Victoria, BC Canada; 6 SABI Mind, Calgary, AB, Canada

**Keywords:** Medical cannabis, PTSD, Plant medicine

## Abstract

The last few decades have seen increasing interest in the use of cannabis for post-traumatic stress disorder (PTSD). Recent attempts to evaluate the clinical efficacy of cannabis for PTSD were inconclusive and generalizability was limited by undesirable features of the study drug. The present clinical trial evaluated the effects of a commercially available chemovar that was delivered by vaporization. The study was designed as a randomized placebo-controlled cross-over study with three conditions; however, only five individuals completed the trial, and analysis of the placebo effect was not possible. Results identified positive changes consistent with medium-sized within-subject effects for cannabis in the treatment of PTSD. Positive trending results and high patient need mandate future studies of cannabis for the treatment of PTSD.

## Introduction

Cannabis is a flowering plant valued for millennia due to its medicinal and psychoactive properties. Interest in the therapeutic application of cannabinoids has re-emerged after decades of marginalization, with the treatment of mental health conditions among the most prominent applications [1]. Post-traumatic stress disorder (PTSD) is a condition for which cannabinoids have demonstrated therapeutic potential, likely through the facilitation of sleep, inhibition of nightmares, and reductions in hyperarousal. Several governments support cannabis access for military veterans, and individuals with PTSD report high levels of medical cannabis use [1].

Preclinical studies implicate the endocannabinoid system in the etiology of PTSD [2], and some positive outcomes have been identified among individuals with PTSD who use cannabis [3]. However, barriers to trialing a product that is widely available to the public and tightly restricted for clinical research has impeded investigations [4]. The lone randomized control trial of smoked cannabis for PTSD was inconclusive, with drug effects muted by a strong placebo response, and generalizability limited by undesirable features of the study drug such as reported harshness and low potency relative to commercially available cannabis, thus leading to calls for further research [5]. The present study was designed to parallel aspects of that trial but used cannabis chemovars that were equivalent to those common to the legal medical cannabis market, and the cannabis was delivered by vaporization rather than combustion. Low power due to under-recruitment prevented the planned placebo-controlled analysis; however, given the dearth of clinical trial research on cannabis for PTSD, our results warrant consideration.

## Methods

**Fig. 1:**
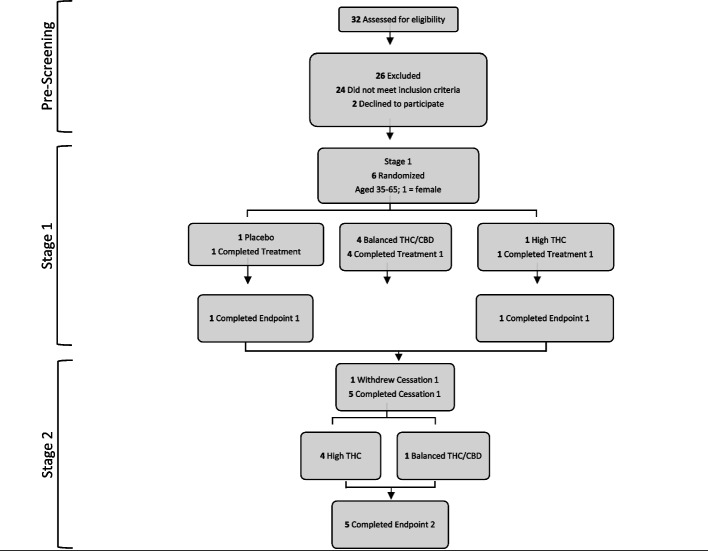
Consort flow design. Note: Stages 1 and 2 include 2 weeks of washout/cessation followed by 3 weeks of ad lib cannabis use

This trial was registered on ClinicalTrials.gov (NCT02517424) and approved by the Clinical Research Ethics Board at the University of British Columbia (H16-01026). Participants provided written informed consent prior to participating. The study was designed as a randomized blinded placebo-controlled cross-over study with three conditions: placebo cannabis (< 1% THC and < 1% CBD), cannabis with 10 + / − 2% THC and 10 + / − 2% CBD, and cannabis with 10 + / − 2% THC and < 1% CBD. These chemovars were selected to allow for a proposed comparison between a psychoactively inert product and cannabis products that varied in CBD but were equivalent in THC. The cross-over design was selected in an attempt to efficiently allow comparisons of all three groups while providing access to one of the two active study drug conditions for all participants. Participants were provided a portable cannabis vaporizer and two grams of cannabis per day for three weeks of ad lib use. Target recruitment was for 42 participants. Thirty-two individuals were assessed for eligibility; 24 did not meet inclusion criteria and two declined to participate. Failure to meet our recruitment target resulted in the study terminating with only six participants (age range 35–65; 1 female) enrolled of whom five completed the entire protocol. Due to low power, we revised our analysis to aggregate active conditions and test within-subject change from baseline to the end of active treatment. The primary outcome was a change in scores on the Clinician Administered PTSD Scale (CAPS-5) [6], and the secondary outcome measure was scores on the PTSD Checklist for DSM-5 (PCL-5) [7]. Total PCL-5 scores were prorated for two participants missing one item. Random assignment placed five participants in an active condition for Phase 1; four received balanced THC/CBD cannabis and one received the THC dominant chemovar (Fig. 1). Baseline measures for these participants were compared to the end of week three. One participant received placebo in Phase 1 and THC cannabis in week six; for this participant, baseline was week five and outcome was week eight. All analyses used paired samples t-tests, with a significance level *p* < 0.05.

## Results

**Fig. 2:**
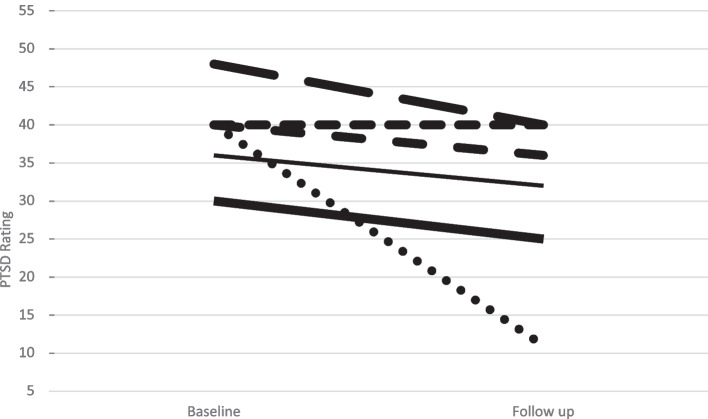
Patient-level change in total PTSD ratings on CAPS-5. Note: CAPS-5, Clinician Administered PTSD Scale for DSM-5; potential range = 0–80

Comparison of CAPS scores pre and post treatment identified a trend toward reduction in PTSD symptoms (*M* = 39.00, *SD* = 5.90 vs *M* = 30.67, *SD* = 11.17); *t*(5) = 1.95, *two-tailed p* = 0.11; *one-tailed p* = 0.06; *d* = 0.80 (Fig. 2). Supplementary analyses of PCL identified a similar effect that more closely approached significance (*M* = 63.93, *SD* = 10.91 vs *M* = 50.61, *SD* = 19.84); *t*(5) = 2.50, *two-tailed p* = 0.05; *one-tailed p* = 0.03; *d* = 1.02.

## Discussion

We identified positive changes consistent with medium-sized within-subject effects for cannabis in the treatment of PTSD. These results are consistent with preclinical and cross-sectional research [1]. However, under-recruitment resulted in low power and prohibited placebo comparison, making these results more suggestive than persuasive. Recruitment difficulties may be partly attributable to the ubiquity of cannabis access in Canada, including legal non-medical adult access, as well as government-subsidized access for veterans with an approved medical need. Participant burden in this complex study may have also deterred participants. Positive trending results and high patient need mandate future studies of cannabis for the treatment of PTSD. These studies should be pragmatic, avoid overly strict inclusion criteria, consider incentivizing participation, and minimize participant burden, particularly if recruiting in a region with easy access to cannabis outside of clinical trials.

## Data Availability

The datasets used and/or analyzed during the current study are available from the corresponding author on reasonable request.
